# Ionomycin suppresses cancer cell growth by disrupting mitochondrial transcription

**DOI:** 10.1002/2211-5463.70297

**Published:** 2026-09-01

**Authors:** Lishen Wang, Mingqiang Deng, Dongmei He, Diqin Lai, Dongjun Liu, Dengyong Zhu, Zhenhua Zhang, Peng Tang, Chuanman Zhou, Menghui Yin, Xichen Bao

**Affiliations:** ^1^ Biomedical Sciences College & Shandong Medicinal Biotechnology Centre Shandong First Medical University & Shandong Academy of Medical Sciences, NHC Key Laboratory of Biotechnology Drugs, Shandong Key Laboratory of Genetic Engineering and Synthetic Biology Jinan China; ^2^ Center for Cell Lineage Atlas Guangzhou Institutes of Biomedicine and Health, Chinese Academy of Sciences China; ^3^ Department of Basic Research Guangzhou National Laboratory China; ^4^ University of Chinese Academy of Sciences Beijing China; ^5^ State Key Laboratory of Respiratory Disease Guangzhou Institutes of Biomedicine and Health, Chinese Academy of Sciences China; ^6^ Guangdong Provincial Key Laboratory of Stem Cell and Regenerative Medicine, Guangdong‐Hong Kong Joint Laboratory for Stem Cell and Regenerative Medicine Guangzhou Institutes of Biomedicine and Health, Chinese Academy of Sciences China; ^7^ Guangzhou Medical University China

**Keywords:** cell proliferation, ionomycin, mitochondrial gene transcription, OXPHOS

## Abstract

The Warburg effect has long suggested that oxidative phosphorylation (OXPHOS) is dispensable for tumor growth. However, recent studies have shown that the mitochondrial RNA polymerase inhibitors IMT1 and IMT1b, which impair OXPHOS, are potent anticancer agents. Here, we demonstrate that ionomycin, a selective ionophore known to modulate mitochondrial homeostasis, similarly inhibits mitochondrial gene expression across cancer cell lines. Specifically, gene expression and nascent RNA profiling revealed a global downregulation of mitochondrial gene transcription in Jurkat T, THP‐1, HeLa, and NCI‐H441 cells. Thus, we conclude that ionomycin suppressed mitochondrial gene transcription, impaired OXPHOS, and thereby inhibited cancer cell proliferation and growth, providing a novel insight into the function of ionomycin.

## Graphical abstract

In this study, we identify a new function for the selective Ca^2+^ ionophore, ionomycin, as an inhibitor of mitochondrial transcription. Both total and nascent RNA analyses revealed that ionomycin treatment reduces the transcription of mitochondrial genes. Thus, ionomycin treatments lead to a decrease in oxidative phosphorylation levels and ultimately impair cancer cell proliferation.

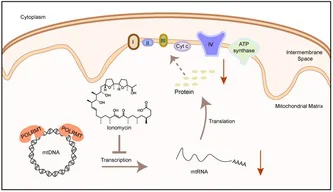

**Abbreviations**
5‐EU: 5‐ethynyl uridine
Act D: actinomycin D
ATP: adenosine triphosphates
CRC: colorectal cancer
DRP1: dynamin‐related protein 1
GO: gene ontology
GSEA: gene set enrichment analysis
IGV: integrative genomics viewer
MCU: mitochondrial calcium uniporter
OCR: oxygen consumption rate
OXPHOS: oxidative phosphorylation
PMA: Phorbol 12‐myristate 13‐acetate
POLRMT: RNA polymerase mitochondrial
ROS: reactive oxygen species
TFAM: transcription factor A
TFB2M: transcription factor B2
VDAC: Voltage‐Dependent Anion Channel

Mitochondria are powerhouses of eukaryotic cells, providing more than 90% of cellular adenosine triphosphates (ATPs) production [1]. In recent years, mitochondria, as essential organelles, have been found to participate in the regulation of diverse physiological and pathological processes, such as cell survival, proliferation, migration, as well as tumor initiation, progression, and metastasis [2]. Growing evidence has demonstrated that abnormalities in glucose, fatty acid, and amino acid metabolism are widespread during tumorigenesis [3]. In addition, mitochondria serve as the cellular factories for the generation of reactive oxygen species (ROS), and dysregulation of ROS promotes malignant tumor progression [4]. Mitochondria are key regulators of apoptosis and can enhance the anti‐apoptotic capacity of cancer cells, thereby accelerating their rapid proliferation [5].

Mitochondria act as a center integrating Ca^2+^ signals into metabolic outputs [6, 7]. Mitochondrial Ca^2+^ uptake is a tightly regulated process involving sequential transfer through the Voltage‐Dependent Anion Channel (VDAC) on the outer membrane and the Mitochondrial Calcium Uniporter (MCU) complex on the inner membrane, with MCU serving as the rate‐limiting and highly selective gateway controlling matrix Ca^2+^ levels [6, 8]. It has long been established that mitochondrial Ca^2+^ accumulation is associated with apoptosis [9]. Under certain stimuli or stress conditions, elevated matrix Ca^2+^ promotes the opening of the relatively nonselective mitochondrial permeability transition pore, leading to the loss of mitochondrial membrane integrity and subsequent release of pro‐apoptotic factors from the mitochondria [10]. Ionomycin, in particular, is a selective ionophore agent [11]. The molecule transports Ca^2+^ across cell membranes and increases the intracellular calcium levels quickly [12]. The increased Ca^2+^ in cells triggers the following calcium signals and is often paired with phorbol 12‐myristate 13‐acetate (PMA), which activates immune cells such as T cells [12, 13]. Previous studies have shown that in colorectal cancer (CRC) cells, mitochondrial Ca^2+^ can regulate the phosphorylation level of the transcription factor TFAM, thereby affecting the transcriptional activity of mitochondrial genes [14]. Earlier studies have shown that ionomycin induced actin cytoskeleton disruption in human osteosarcoma U2OS cells, which stimulated dynamin‐related protein 1 (Drp1) oligomerization and subsequent mitochondrial fission [15]. In human large cell lung carcinoma cells (LCLC 103H), ionomycin elevated intracellular Ca^2+^ levels and activated calpain. Activated calpain then cleaved BCL‐2 family members (Bcl‐2, Bid, Bcl‐xL) and disrupted their function, ultimately triggering mitochondrial cytochrome c release and the intrinsic apoptotic pathway [16].

RNA polymerase mitochondrial (POLRMT) is the key enzyme responsible for mtDNA transcription in eukaryotic cells [17, 18]. After being transported into mitochondria, it synthesizes mitochondrial RNA molecules required for the production of critical components of respiratory chains [19, 20]. Therefore, POLRMT is crucial for cellular energy metabolism, due to its role in synthesizing components that are indispensable for mitochondrial energy production [21]. POLRMT plays an important role in the initiation of mitochondrial transcription. Concretely, it acts in concert with the transcription factors TFAM and TFB2M [18, 22].

Maintaining normal mitochondrial function and mtDNA integrity is essential for tumor cell survival and progression, as they regulate key processes such as energy metabolism, ROS production, and genomic stability. Consistent with this, widespread dysregulation of mitochondrial genes has been reported in various cancers. Tumor cells are characterized by elevated glycolytic activity compared with normal cells, suggesting they require more energy [23, 24]. Interestingly, recent studies have revealed that oxidative phosphorylation (OXPHOS) is upregulated to sustain metabolic requirements in tumors, such as acute myeloid leukemias, lymphomas, and endometrial carcinoma cells [25]. In addition, the inhibition of POLRMT is associated with tumor suppression, suggesting a way to identify anticancer agents by repressing transcription in mitochondria [26]. For instance, IMT1 and IMT1b suppress transcription of mitochondria‐encoded OXPHOS subunits, such as *ND 1–6* of complex I and *COX I‐IV* of complex IV, thereby inactivating cancer cell metabolism. Of note, IMT1 has been demonstrated to exert antitumor effects across multiple cancer cell lineages [27, 28, 29]. This observation indicates that inhibiting mitochondrial gene expression, thereby reducing oxidative phosphorylation in tumor cells, may constitute a novel therapeutic approach for cancer treatment.

RNA sequencing (RNA‐seq), which primarily profiles mature mRNA as the final transcriptional output, provides limited insight into the dynamic regulation of transcription. In contrast, sequencing‐based nascent RNA approaches enable direct measurement of ongoing RNA synthesis and capture short‐lived transcripts, thereby offering a powerful tool to investigate transient transcriptional changes induced by short‐term stimuli [30, 31, 32]. Moreover, large‐scale integration of nascent RNA datasets has led to the development of several databases that systematically characterize genome‐wide transcriptional activity and its dynamics [33]. These approaches offer critical advantages for investigating transcriptional regulation in specific contexts, such as the inhibition of mitochondrial gene transcription by ionomycin.

In this study, we applied nascent and total RNA analyses to investigate transcriptional alterations in mitochondria following ionomycin treatment. We also systematically explored the impacts of ionomycin on cancer cell growth using multiple canonical cancer cell lineages (Jurkat T, HeLa, NCI‐H441, and THP‐1 cells). We found that ionomycin inhibits mtDNA transcription in cancer cells and results in impaired OXPHOS activity, and consequently suppresses tumor cell proliferation. Collectively, these findings demonstrate the potential application of ionomycin in controlling cancer cells. This may also introduce a novel therapeutic strategy to suppress cancer cell progression via targeting OXPHOS.

## Materials and methods

### Cell culture

HeLa (RRID: CVCL_0030) and NCI‐H441 (RRID: CVCL_1561) cells were cultured in DMEM (L120KJ; BasalMedia) supplemented with 10% fetal bovine serum (S711‐001S; LONSERA) and 1% penicillin–streptomycin (SV30010; HyClone). Jurkat T (RRID: CVCL_0065) and THP‐1 (RRID: CVCL_5115) cells were cultured in RPMI‐1640 medium (L210KJ; BasalMedia) containing 10% FBS and 1% penicillin–streptomycin (P/S); THP‐1 cells additionally required 0.5% 2‐mercaptoethanol (21 985 023; Gibco). All cell lines were cultured at 37 °C and 5% CO_2_. Cell lines were derived from an authenticated stock from ATCC, and all cells were tested and confirmed to be free of mycoplasma contamination using the Mycoalert One‐Step Mycoplasma Detector (D201‐02; Vazyme).

### Super‐resolution imaging

Super‐resolution imaging was performed using Zeiss Elyra 7 with Lattice SIM^2^. Images were captured with a 100× oil immersion objective. Single plane images were preprocessed with consistent parameters in SIM^2^.

### Mitochondrial membrane potential assay

Mitochondrial membrane potential (MMP) was assessed using the TMRE‐based assay, as TMRE preferentially accumulates in hyperpolarized mitochondria, and its fluorescence intensity positively correlates with MMP [34]. Briefly, Jurkat T cells were incubated with 1× TMRE (C2001S; Beyotime Biotechnology) for 30 min at 37 °C in the dark. After incubation, cells were washed twice with PBS and resuspended in culture medium. Fluorescence intensity was subsequently analyzed by flow cytometry using a BD LSRFortessa (BD Biosciences).

### Reactive oxygen species (ROS) assay

Mitochondrial reactive oxygen species (mtROS) levels were measured using MitoSOX™ Red (M36007; Thermo Fisher Scientific). Cells were incubated with MitoSOX™ Red reagent at 37 °C for 15 min in the dark, washed twice with PBS, and resuspended in culture medium. Fluorescence signals were detected by flow cytometry using a BD LSRFortessa.

### Mitochondrial Ca^2+^ assay

Mitochondrial calcium levels were measured using the fluorescent indicator Rhod‐2 AM (S1062S; Beyotime Biotechnology). Cells were incubated with 2 μm Rhod‐2 AM at 37 °C for 30 min, washed twice with PBS, and resuspended in culture medium. Fluorescence signals were detected by flow cytometry using a BD LSRFortessa.

### Cell cycle analysis

For cell cycle determination, cells were fixed, stained with propidium iodide (C1008M; Beyotime Biotechnology), and then analyzed on the BD LSRFortessa flow cytometer. DNA content was measured to determine the distribution of cells in G0/G1, S, and G2/M phases.

### Nascent RNA sequencing

Nascent RNA sequencing was conducted as previously described [35]. Nascent transcripts were metabolically labeled with 5‐ethynyl uridine (5‐EU), and total RNA was extracted using TRNzol Universal (DP424; TIANGEN) according to the manufacturer's instructions. RNA concentration and purity were measured with a NanoPhotometer N60 (IMPLEN). A total of 200 μg of RNA was subjected to a click chemistry reaction (100 μm biotin‐azide, 500 μm CuSO_4_, 2 mm THPTA, and 5 mm vitamin C) for biotinylating, followed by fragmentation using NaOH. Dynabeads™ MyOne™ Streptavidin C1 (65001; Invitrogen) were then used for RNA pull‐down. After sequencing adapters were ligated, cDNA was synthesized, and the library was sequenced on the Illumina platform.

### RNA isolation and quantitative reverse transcription polymerase chain reaction (RT‐qPCR)

Total RNA was extracted using TRNzol Universal RNA Reagent (DP424; TIANGEN), cDNA was synthesized with HiScript IV RT SuperMix (R423‐01; Vazyme). Quantitative RT‐qPCR was performed in QuantStudio™ 3 real‐time quantitative PCR instrument (Thermo Fisher) following the protocol of initial denaturation at 95 °C for 30 s, 40 cycles of 95 °C for 5 s, 60 °C for 30 s. Gene expression was normalized to 18S and calculated via the 2^−ΔΔCt^ method. All primers for qPCR were listed in Table 1.

**Table 1:** Specific primers for qPCR.

Gene	Primer ID sequence (5′–3′)
*RNR1*	F: TAGAGGAGCCTGTTCTGTAATCGAT R: CGACCCTTAAGTTTCATAAGGGCTA
*RNR2*	F: ACCAGACGAGCTACCTAAGA R: CTTGGACAACCAGCTATCAC
*COX3*	F: TTCACCATTTCCGACGGCAT R: GGCGGATGAAGCAGATAGTGA
*ATP6*	F: CGCCGCAGTACTGATCATTCT R: TCGGTTGTTGATGAGATATTTGGA
*CYTB*	F: TCTCCGATCCGTCCCTAACA R: GTGATTGGCTTAGTGGGCGA
*18S*	F: GTAACCCGTTGAACCCCATT R: CCATCCAATCGGTAGTAGCG

### EU pulse–chase assay for RNA stability

RNA stability was assessed using a 5‐ethynyl uridine (EU) pulse–chase assay [36]. Jurkat T cells were treated with the nucleoside analog 5‐ethynyluridine (EU) for 16 h to label transcribed RNA, followed by Actinomycin D treatment for 2 h to inhibit RNA transcription. Cells were then either treated with ionomycin (2 μg·mL^−1^) for 16 h or treated with DMSO. Total RNA was then extracted and subjected to biotinylation via click chemistry to selectively capture EU‐labeled RNA. The labeled RNA fraction was isolated by streptavidin‐mediated pull‐down, and the abundance of mitochondrial transcripts was quantified by qPCR.

### Functional annotations and enrichment analysis

To annotate the enriched functions of differentially expressed genes following phorbol 12‐myristate 13‐acetate (PMA) and ionomycin cotreatment or ionomycin treatment alone, Gene Ontology (GO) enrichment analysis was performed in Metascape using default parameters. Gene Set Enrichment Analysis (GSEA) was also conducted, with the oxidative phosphorylation (OXPHOS) pathway gene set obtained from MSigDB [37]. Statistical significance for GSEA was determined using logistic regression with a *P* value < 0.05.

### Nascent RNA‐qPCR

Nascent transcripts were metabolically labeled with 1 mm 5‐EU for 15 min, and total RNA was isolated using TRNzol Universal (DP424; TIANGEN) according to the manufacturer's instructions. RNA concentration and purity were measured with a NanoPhotometer N60, after which 20 μg of RNA was biotinylated via click chemistry. Biotin‐labeled RNA was captured using Dynabeads™ MyOne™ Streptavidin C1, followed by direct cDNA synthesis. Quantification was subsequently performed using the QuantStudio™ 3 real‐time quantitative PCR instrument.

### ATP measurement

Intracellular ATP was measured using an ATP Assay kit (S0026; Beyotime Biotechnology) according to the manufacturer's instructions. Luminescence was measured using a GloMax® Discover microplate reader (Promega), and the concentration of ATP was calculated using an ATP standard curve.

### Oxygen consumption rate (OCR)

Jurkat T cells were seeded in black‐walled, clear‐bottom 96‐well plates at a density of 5 × 10^3^ cells per well. Then, the OCR assay working solution was added according to the manufacturer's instructions (E‐BC‐F070; Elabscience). The plate was loaded into a microplate reader maintained at 37 °C, with measurements taken every 2 min for 90 min. Fluorescence values were plotted against time, and the linear phase of the curve was selected for OCR quantification based on the slope value.

### Western blotting

Total protein extraction from Jurkat T cells was performed using RIPA buffer (P0013C; Beyotime Biotechnology) for cell lysis, followed by protein concentration quantification with a Pierce BCA Protein Assay Kit (RK240545; Thermo). Separated using 10% SDS/polyacrylamide gel electrophoresis, the membranes were blocked for 1 h at room temperature with 5% milk in TBST (0.1% Tween‐20 in tris‐buffered saline), and then incubated overnight at 4 °C with the primary antibodies. The membranes were subsequently washed with TBST before incubating with a secondary antibody for 1 h at room temperature. The bands were visualized using a chemiluminescence kit (WBKLS0500; Millipore). All antibodies for western blotting were listed in Table 2.

**Table 2:** Details of antibodies used for studying.

Antibody	Product code	Manufacturer
MFN1	13 798‐1‐AP	Proteintech
DRP1	ab184247	Abcam
Phospho‐DRP1‐S616	ab314755	Abcam
p62	ab56416	Abcam
NDUFB8	14 794‐1‐AP	Proteintech
VDAC	ab154856	Abcam
Calnexin	2679S	Cell Signaling Technology
ATP6	55 313‐1‐Ap	Proteintech
COX IV	4844	Cell Signaling Technology
Anti‐mouse secondary antibody	7076S	Cell Signaling Technology
Anti‐rabbit secondary antibody	7074S	Cell Signaling Technology

### CCK‐8 assays

Log‐phase cells were seeded in 96‐well plates at a density of 2 × 10^3^ cells per well with 200 μL complete medium (10% FBS + 1% penicillin–streptomycin). At designated time points (0 h, 24 h, 48 h), 10 μL of CCK‐8 solution (K1018; APeXBIO) was added to each well without bubble formation. Plates were further incubated for 1 h (optimized based on preliminary tests) until color development stabilized. OD values were measured at 450 nm using a GloMax® Discover microplate reader. Data were presented as mean ± SD, and proliferation curves were analyzed by GraphPad Prism 9.0.

### Colony formation assay

NCI‐H441 and HeLa cells were seeded in 6‐well plates with complete culture medium, followed by treatment with or without ionomycin. After 2 weeks of incubation at 37°C under 5% CO_2_, cells were gently washed once with PBS, fixed with 4% paraformaldehyde (AR1068; BOSTER) for 15 min at room temperature, and stained with 0.1% crystal violet for 30 min. Colonies were photographed and quantified using photography equipment (Colony Formation Assay). Three independent biological replicates were performed, and data are presented as mean ± SD.

### Soft agar colony formation assay

Each well of a 6‐well plate was precoated with 1 mL of 0.6% agar (in complete medium) and solidified at room temperature. Jurkat T and THP‐1 cells (2 × 10^3^ per well) were resuspended in 2 × complete medium (20% FBS) and mixed 1:1 with 0.6% agarose (final concentration: 0.3%). 0.1 mL of cell‐agarose mixture was gently overlaid onto the presolidified base layer. Plates were kept horizontally for 15 min at room temperature to solidify. Top layers were supplemented with 2 × complete medium containing 2 μg·mL^−1^ ionomycin or vehicle control. Plates were incubated at 37 °C under 5% CO_2_ for 3 weeks, with medium replacement every 3 days. Colonies were stained with 0.1% crystal violet for 10 min, photographed under a microscope, and counted [38].

### Statistical analysis

All data were analyzed using the licensed graphpad prism software and are presented as mean ± standard deviation (SD). Differences between two groups were assessed by multiple *t*‐tests with correction. A *P*‐value < 0.05 was considered statistically significant (*P < 0.05, **P < 0.01, ***P < 0.001, ****P < 0.0001).

### Data visualization

Bedgraphs and bigwig format were obtained for visualization in Integrative Genomics Viewer (IGV). Data were analyzed in RStudio and visualized using the ggplot2 library and R packages pheatmap and ComplexHeatmap.

## Results

### Ionomycin treatment reduces mitochondrial gene expression

**Fig. 1:**
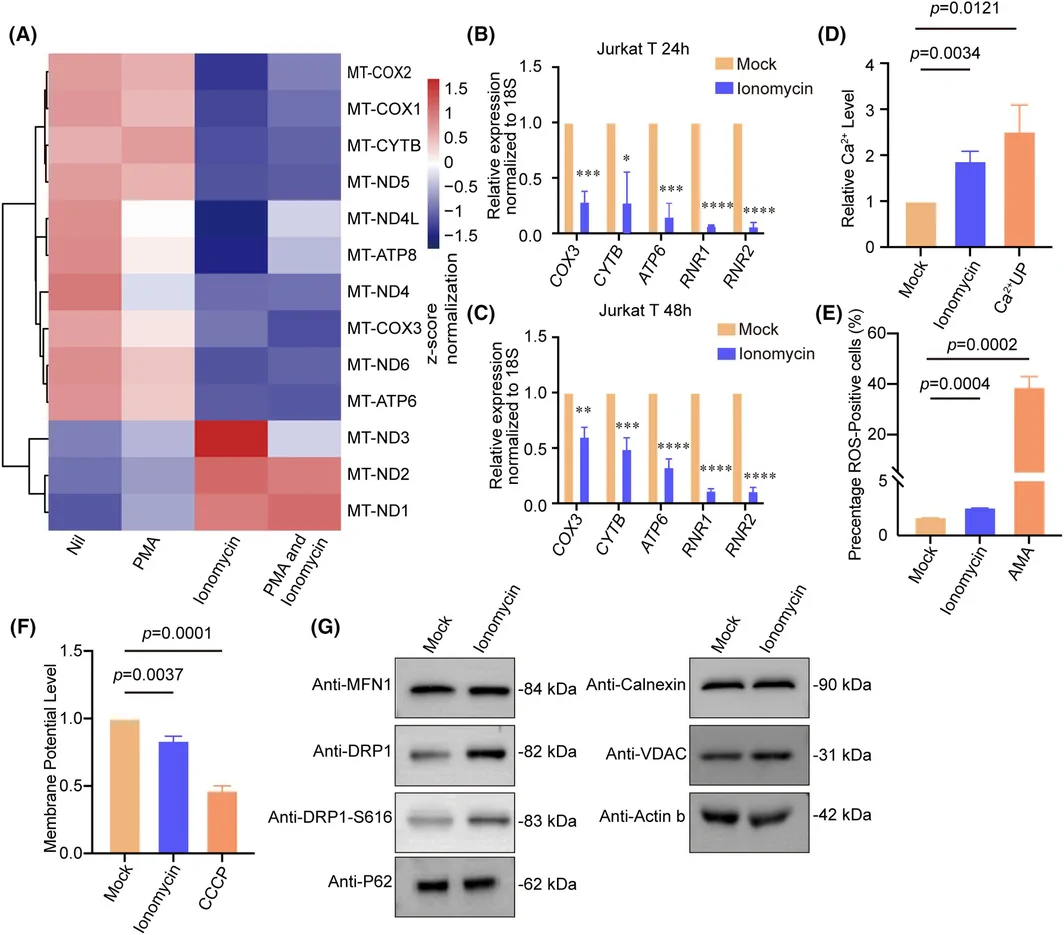
Ionomycin reduces mitochondrial gene expression and disrupts mitochondrial homeostasis in human T lymphoblastic leukemia cells (Jurkat T cells). (A) Published Transcriptomic data indicating changes in mitochondrial gene expression levels in Jurkat T cells following ionomycin (2 μg·mL^−1^) treatment. (B, C) Total‐qPCR analysis of mitochondrial‐encoded rRNAs (*RNR1, RNR2*) and OXPHOS complex genes (*CYTB, ATP6, COX3*) in Jurkat T cells, after treatment with ionomycin (2 μg·mL^−1^) for 24 h and 48 h. (mean ± SD, *n* = 3 biological replicates, two‐sided Student's *t*‐test, *P < 0.05, **P < 0.01, ***P < 0.001, ****P < 0.0001). (D) Quantification of mitochondrial Ca^2+^ levels shown as Mean Fluorescence Intensity (MFI) from. Mitochondrial Ca^2+^ levels in Jurkat T cells treated with ionomycin (2 μg·mL^−1^) for 2 h, measured by flow cytometry. Ca^2+^ UP was used as a positive control. (E) Quantification of the percentage of mtROS‐positive cells from. Mitochondrial ROS production in Jurkat T cells following ionomycin (2 μg·mL^−1^) treatment for 2 h, measured by flow cytometry. Treatment with Antimycin A (AMA, 50 μg·mL^−1^) for 0.5 h was used as a positive control. (F) Quantification of mitochondrial membrane potential shown as MFI from. Mitochondrial membrane potential in Jurkat T cells following ionomycin (2 μg·mL^−1^) treatment for 2 h, measured by flow cytometry. Treatment with Carbonyl cyanide m‐chlorophenyl hydrazone (CCCP, 10 mm) for 0.5 h was used as a positive control. (G) Western blot analysis of mitochondrial dynamics–related markers Mitofusin 1 (MFN1), Dynamin‐related protein 1 (DRP1), and p‐DRP1 (Ser616) in Jurkat T cells treated with ionomycin (2 μg·mL^−1^) for 2 h. Protein levels of the endoplasmic reticulum marker Calnexin, mitochondrial outer membrane protein VDAC, and autophagy marker p62 were also assessed.

In a published RNA‐seq dataset, we observed significantly suppressed expression of mitochondrial genes in Jurkat T cells [39] (Fig. 1A). To further validate the observation, we profiled the transcriptional changes of mitochondrial genes via qPCR in Jurkat T cells that were treated with ionomycin. The results show that under ionomycin treatment (2 μg·mL^−1^), the expression levels of mitochondrial genes significantly decreased, such as *MT‐ATP6* (Fig. 1B, C).

### Ionomycin disrupts mitochondrial homeostasis in cancer cells

Considering the role of ionomycin as a Ca^2+^ ionophore, we first measured mitochondrial Ca^2+^ levels in Jurkat T cells treated with ionomycin (2 μg·mL^−1^) for 2 h. As expected, ionomycin indeed increases cytomatrix Ca^2+^ uptake and elevates mitochondrial Ca^2+^ levels (Fig. 1D and Fig. S1A).

Specifically, we explored the effect of ionomycin on mitochondrial homeostasis. We initially examined the effects of ionomycin on mitochondrial ROS production and mitochondrial membrane potential. In Jurkat T cells, treatment with ionomycin (2 μg·mL^−1^) for 2 h increased ROS levels, but the increase was significantly lower than that caused by antimycin A (AMA, an inhibitor of mitochondrial complex III) [40] (Fig. 1E and Fig. S1B). Ionomycin (2 μg·mL^−1^) treatment for 2 h reduced the mitochondrial membrane potential in Jurkat T cells, similar to carbonyl cyanide m‐chlorophenyl hydrazone (CCCP, a mitochondrial uncoupler) [41] (Fig. 1F and Fig. S1C). We then examined the impact of ionomycin on mitochondrial fusion and fission. Jurkat T cells were treated with ionomycin for 2 h, and the expression of mitochondrial dynamics markers was analyzed. Consistent with previous reports [42, 43], mitochondrial fusion marker Mitofusin 1 (MFN1) showed no significant change. In contrast, the fission marker dynamin‐related protein 1 (DRP1) and its phosphorylated form p‐DRP1 (Ser616) were significantly upregulated (Fig. 1G). These results suggest that short‐term ionomycin treatment promotes mitochondrial fission. Previous studies in human U2OS cells have shown that ionomycin can lead to a ‘pearling’ mitochondrial phenotype [44]. Furthermore, using SIM^2^ super‐resolution imaging, we examined changes in mitochondrial morphology. After ionomycin (2 μg·mL^−1^) treatment for 2 h or 24 h, mitochondria in Jurkat T cells appeared aggregated into punctate structures, with a more pronounced effect observed at 24 h (Fig. S2A). In addition, ionomycin (2 μg·mL^−1^) treatment for 2 h did not significantly affect mtDNA content (Fig. S2B), and the protein levels of the autophagy marker p62 were not significantly changed (Fig. 1G), suggesting no significant impact on mitophagy.

In addition, we sought to investigate whether ionomycin affects other organelles. We also examined whether it modulates genes involved in mitochondrial calcium regulation. The expression levels of calnexin (an endoplasmic reticulum protein) and VDAC (a mitochondrial outer membrane protein involved in Ca^2+^ transport) after ionomycin (2 μg·mL^−1^) treatment for 2 h showed no significant changes (Fig. 1G). Finally, we further assessed the effect of ionomycin on the cell cycle of Jurkat T cells. Flow cytometry analysis showed that 2 h treatment did not significantly alter cell cycle distribution (Fig. S2C).

### Ionomycin treatment reduces mitochondrial transcript levels without affecting mitochondrial RNA stability

**Fig. 2:**
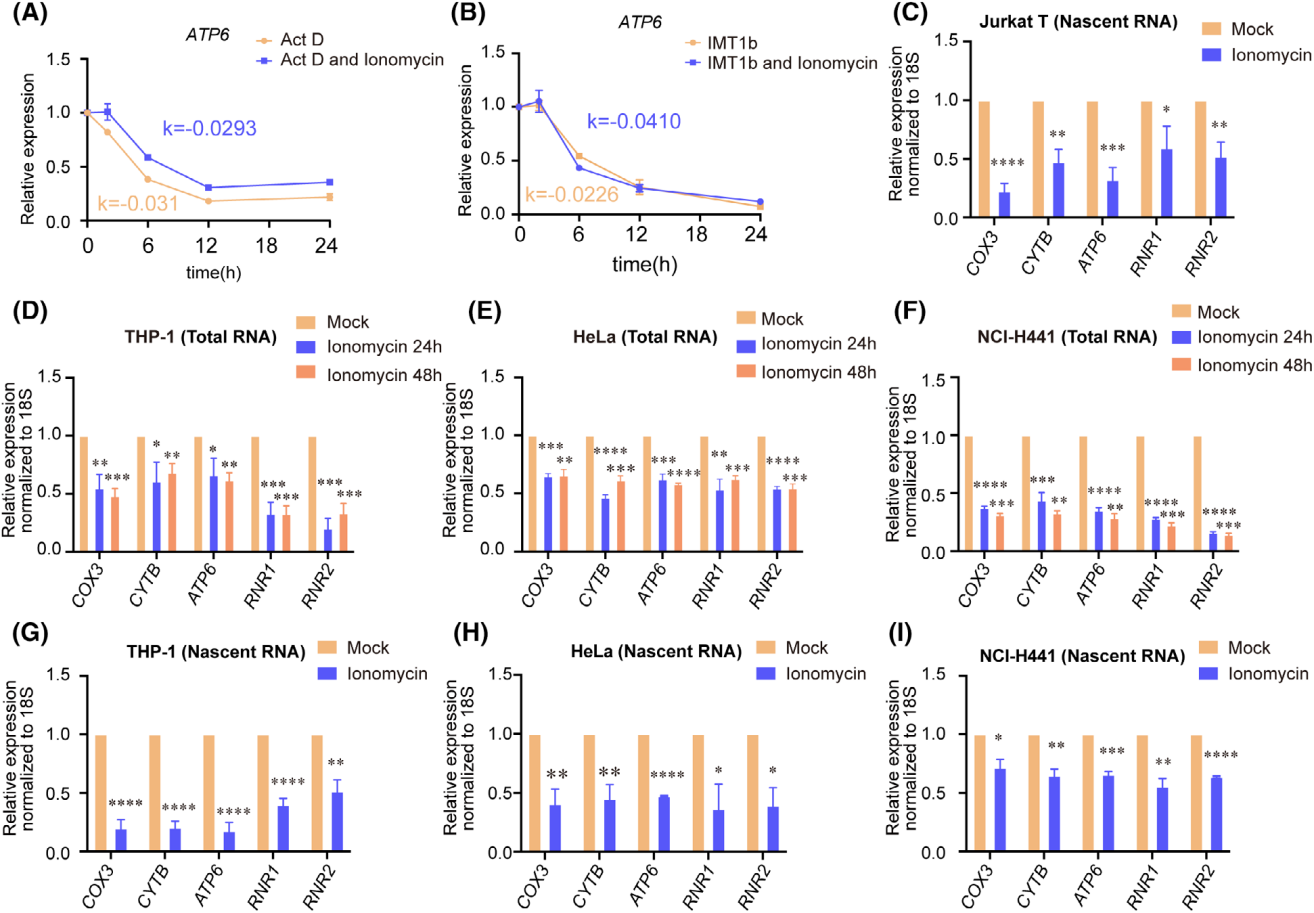
Ionomycin exhibits broad‐spectrum inhibition of mitochondrial gene transcription. (A) Following inhibition of cellular transcription with Actinomycin D (Act D, 10 μg·mL^−1^), the effect of ionomycin on the levels of mitochondrial genes *ATP6* was evaluated. (B) Following inhibition of mitochondrial transcription with the mitochondrial transcription inhibitor IMT1b (1 μg·mL^−1^), the effect of ionomycin on the levels of mitochondrial genes *ATP6* was assessed. (C) Nascent RNA‐qPCR results in Jurkat T cells. (D–F) Mitochondrial gene expression in human monocytic leukemia cells (THP‐1) (D), cervical cancer cells (HeLa) (E), and human lung adenocarcinoma cells (NCI‐H441) (F) following ionomycin (2 μg·mL^−1^) treatment for 24 h and 48 h. (G–I) Nascent RNA‐qPCR analysis of THP‐1 (G), HeLa (H), and NCI‐H441 (I) cells treated with ionomycin (2 μg·mL^−1^) for 2 h (mean ± SD, *n* = 3 biological replicates, two‐sided Student's *t*‐test, *P < 0.05, **P < 0.01, ***P < 0.001, ****P < 0.0001).

To investigate the underlying mechanisms by which ionomycin regulates mitochondrial gene expression, we first examined mitochondrial RNA stability after inhibition of transcription with Actinomycin D (Act D) [45] or IMT1b [26]. The results showed that ionomycin treatment had no significant effect on the stability of mtRNA, including the mitochondrial gene *ATP6* (Fig. 2A,B) and *CYTB* (Fig. S2D,E). Meanwhile, we observed that ionomycin did not significantly affect the stability of RNA labeled with 5‐ethynyl uridine (5‐EU) (Fig. S2F). Supporting this, we applied the 5‐EU‐based nascent RNA assay to monitor newly synthesized RNA (Fig. S2G). Nascent RNA‐qPCR analysis showed that ionomycin treatment for 2 h significantly inhibited mitochondrial RNA transcription (Fig. 2C). Collectively, the mtRNA stability and nascent RNA quantification results reveal that downregulated mitochondrial genes could be attributed to the transcriptional suppression by ionomycin, instead of via a mitochondrial degradation pathway [46, 47]. To assess the generality of ionomycin's effect, we examined additional cancer cell lines (THP‐1, HeLa, and NCI‐H441). Total and nascent RNA analyses in THP‐1 (Fig. 2D,G), HeLa (Fig. 2E,H), and NCI‐H441 (Fig. 2F,I) cancer cell lines, validated by qPCR, demonstrated that ionomycin similarly suppressed mitochondrial gene transcription in these cells, confirming its broad activity across multiple tumor types.

### Global analysis of gene expression following ionomycin treatment by nascent RNA sequencing

**Fig. 3:**
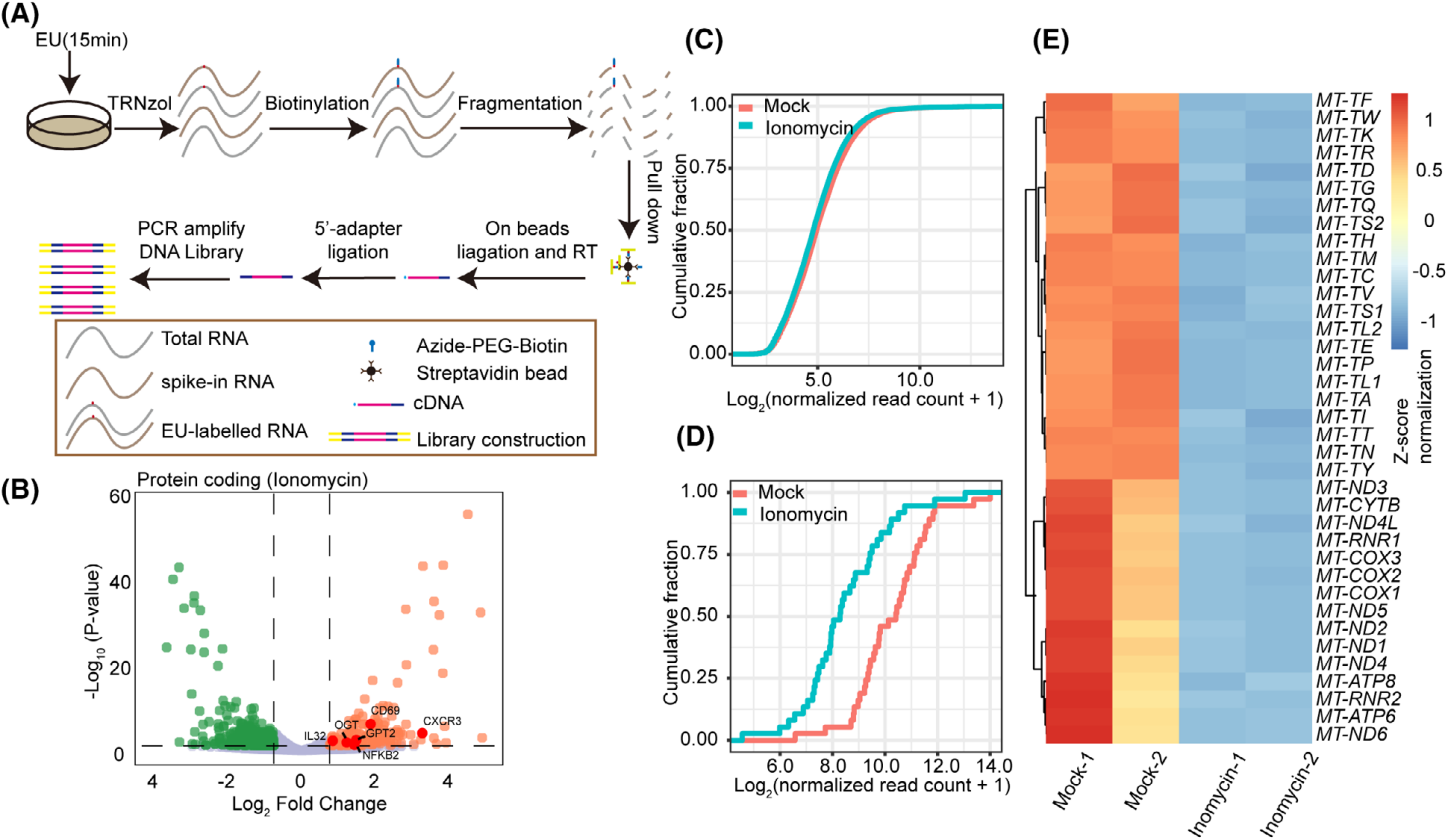
Further validation by nascent RNA‐seq in Jurkat T cells. (A) Schematic diagram of the nascent RNA‐Seq workflow. 5‐EU penetrates the Jurkat T cells membrane and incorporates into newly synthesized RNA without being incorporated into DNA. Through a subsequent click chemistry reaction, biotin containing an azide group is covalently linked to the alkyne group. After RNA fragmentation, nascent RNA is captured using streptavidin‐coated magnetic beads, sequencing adapters are ligated, and PCR amplification is performed prior to next‐generation sequencing. (B) Volcano plot analysis of expression levels of protein‐coding genes in Jurkat T cells following ionomycin (2 μg·mL^−1^) treatment for 2 h (|log_2_ fold change| > 0.585, *p* value < 0.05). (C, D) Empirical cumulative distribution function (ECDF) analysis of protein coding genes (C) and mitochondrial encoded genes (D) based on nascent RNA‐seq data from Jurkat T cells treated with DMSO or ionomycin (2 μg·mL^−1^). Read counts were normalized based on spike‐in controls and then transformed as log_2_ (*x* + 1) before plotting. (E) Heat map analysis of mitochondrial gene expression levels with ionomycin (2 μg·mL^−1^) treatment for 2 h.

To identify transcriptional changes regulated by ionomycin, we performed nascent RNA‐seq (Fig. 3A). Nascent RNA‐seq analysis revealed 102 transcriptionally upregulated and 298 downregulated nuclear‐encoded genes upon ionomycin treatment (|log_2_ fold change| > 0.585, *P* < 0.05). During T‐cell activation, differential expression of marker genes is commonly observed. In our dataset, Jurkat T cells treated with ionomycin displayed altered expression of several activation‐related marker genes, including *OGT* [48], *IL32* [49], *GPT2* [50], *CXCR3* [51] and *NFKB2* [52], indicating changes associated with a T‐cell activation‐like state (Fig. 3B and Table S1). Next, we tested whether the inhibition of transcription caused by ionomycin was specific to mitochondria. Nascent RNA sequencing data showed that, at the global level, ionomycin did not downregulate nuclear‐encoded genes. Instead, it primarily inhibited mitochondrial gene transcription (Fig. 3C,D). To test our hypothesis that ionomycin suppresses the transcription of mitochondrial genes, we next focused on the expression profiles of the mitochondrial genome. The data demonstrated global downregulation of mitochondrial transcription, including mitochondrial‐encoded rRNAs, tRNAs, and mRNAs (Fig. 3E and Table S2).

**Fig. 4:**
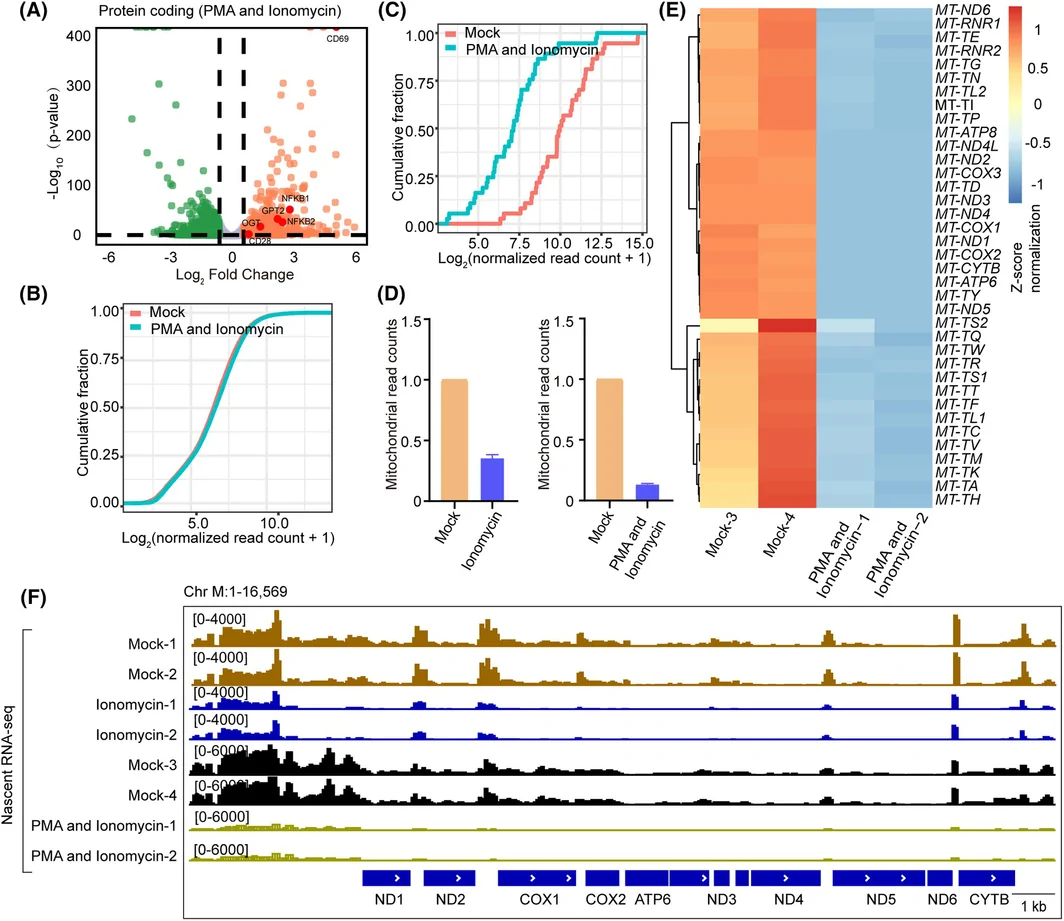
Combined treatment with phorbol 12‐myristate 13‐acetate (PMA) and ionomycin enhances the suppression of mitochondrial gene expression in Jurkat T cells. (A) Volcano plot analysis of expression levels of protein‐coding genes in Jurkat T cells following PMA (50 ng·mL^−1^) and ionomycin (2 μg·mL^−1^) cotreatment for 2 h (|log_2_ fold change| > 0.585, *p* value < 0.05). (B, C) Empirical cumulative distribution function (ECDF) analysis of protein‐coding genes (B) and mitochondrial encoded genes (C) based on nascent RNA‐seq data from Jurkat T cells treated with DMSO or PMA (50 ng·mL^−1^) and ionomycin (2 μg·mL^−1^) co‐treatment for 2 h. Read counts were normalized based on spike‐in controls and then transformed as log_2_ (*x* + 1) before plotting. (D) Bar chart analysis of mitochondrial read counts with ionomycin (2 μg·mL^−1^) treatment for 2 h or PMA (50 ng/mL) and ionomycin (2 μg·mL^−1^) co‐treatment for 2 h. (E) Heat map analysis of mitochondrial gene expression levels with PMA (50 ng·mL^−1^) and ionomycin (2 μg·mL^−1^) cotreatment for 2 h. (F) Integrative Genomic Viewer (IGV) snapshot of mitochondrial gene expression levels following ionomycin (2 μg·mL^−1^) treatment for 2 h and PMA (50 ng·mL^−1^) and ionomycin (2 μg·mL^−1^) co‐treatment for 2 h. The values in square brackets on the y‐axis represent Nascent RNA‐seq read intensity.

Previous studies have shown that phorbol 12‐myristate 13‐acetate (PMA) is commonly used together with ionomycin to stimulate Jurkat T cells [12]. To investigate the specific contribution of PMA during ionomycin‐induced mitochondrial transcriptional suppression, Jurkat T cells were cotreated with PMA and ionomycin. We also examined changes in nuclear‐encoded genes. In contrast to ionomycin treatment alone, cotreatment with PMA and ionomycin resulted in 2131 upregulated and 1518 downregulated nuclear‐encoded genes (|log_2_ fold change| > 0.585, *P* < 0.05). Notably, several T‐cell activation‐associated marker genes, including *CD69* [53], *CD28* [54], *NFKB1* [55], *GPT2* [50], and *OGT* [48], were markedly upregulated under cotreatment conditions (Fig. 4A and Table S3). We also examined the extent of transcriptional changes at the global level for both nuclear and mitochondrial genes. Consistently, cotreatment did not cause a downregulation of nuclear gene transcription (Fig. 4B). The inhibition was restricted to mitochondrial genes (Fig. 4C,E and Table S4). Next, we quantified the changes in mitochondrial read counts under treatment with ionomycin alone or PMA and ionomycin cotreatment. Mitochondrial read counts were lower under cotreatment, indicating a stronger degree of inhibition (Fig. 4D). Moreover, results from Integrative Genomics Viewer (IGV) analysis further confirmed that, compared with ionomycin treatment alone, co‐treatment exerted a stronger inhibitory effect on mitochondrial transcription (Fig. 4F).

In addition, no significant transcriptional changes were observed in rDNA under either ionomycin treatment alone or PMA and ionomycin co‐treatment (Fig. S2H). Meanwhile, PMA treatment alone for 24 h did not induce significant changes in mitochondrial RNA expression in Jurkat T cells (Fig. S2I).

### Ionomycin inhibits transcription of genes involved in oxidative phosphorylation in cancer cells

**Fig. 5:**
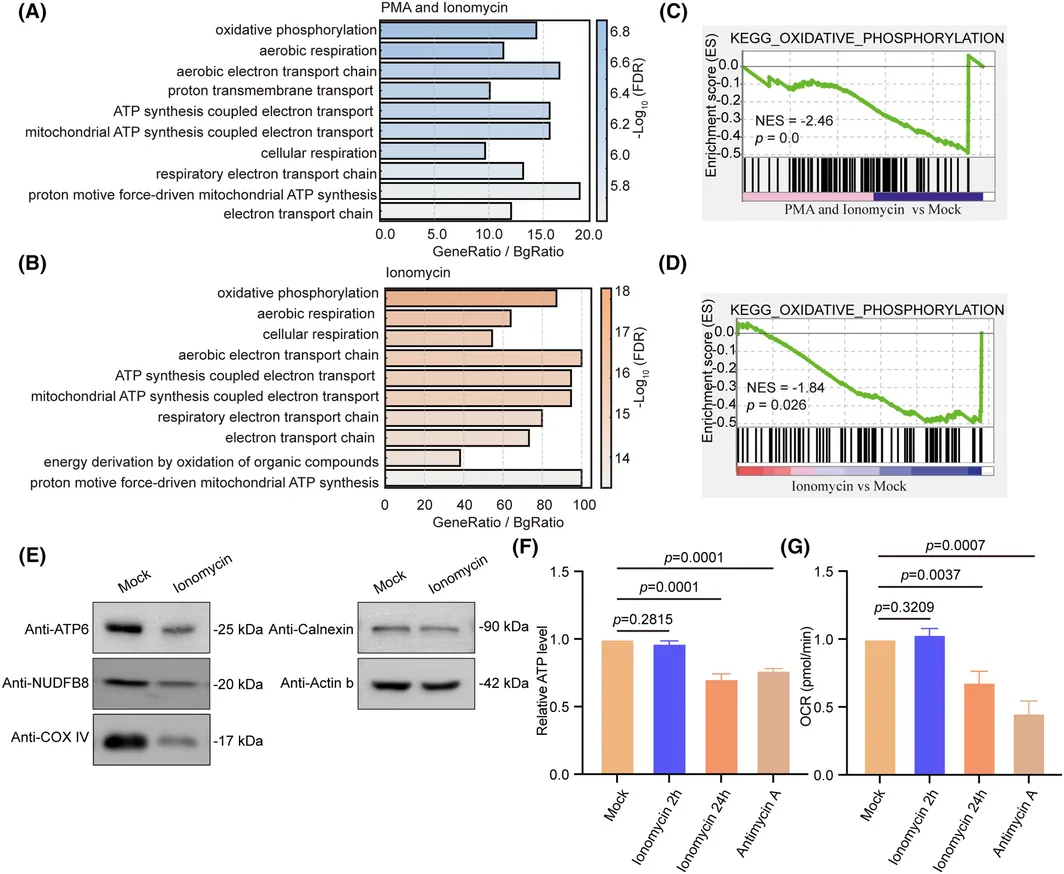
Ionomycin treatment can influence oxidative phosphorylation in Jurkat T cells. (A, B) Gene Ontology (GO) enrichment analysis of downregulated genes in Jurkat T cells with PMA (50 ng·mL^−1^) and ionomycin (2 μg·mL^−1^) cotreatment for 2 h (A) or ionomycin (2 μg·mL^−1^) treatment for 2 h (B) revealed significant enrichment of energy metabolism‐related pathways, including the oxidative phosphorylation pathway. (C, D) Gene Set Enrichment Analysis (GSEA) of oxidative phosphorylation pathway genes from the Kyoto Encyclopedia of Genes and Genomes (KEGG) collections demonstrated statistical significance in PMA (50 ng·mL^−1^) and ionomycin (2 μg·mL^−1^) cotreatment for 2 h (C) or ionomycin (2 μg·mL^−1^) treatment for 2 h (D). (E) Western blot analysis of the effect of ionomycin (2 μg·mL^−1^) treatment for 24 h on protein levels of respiratory chain complex I, IV, and V subunits (NDUFB8, COX IV, and ATP6) and the ER‐associated protein (calnexin). (F, G) Mitochondrial ATP production (F) and oxygen consumption rate (OCR) (G) following treatment with ionomycin (2 μg·mL^−1^) for 2 h or 24 h (mean ± SD, *n* = 3 biological replicates, two‐sided Student's *t*‐test).

Mitochondria possess their own genome encoding key subunits of the oxidative phosphorylation (OXPHOS) system. Dysregulated mitochondrial transcription may lead to metabolic reprogramming in cells, resulting in elevated glycolysis and abnormal expression of OXPHOS components [56, 57]. We next investigated how ionomycin alone or PMA and ionomycin cotreatment affects mitochondrial function in cancer cells. Gene Ontology (GO) analysis of downregulated genes after ionomycin treatment revealed significant enrichment in OXPHOS pathways (Fig. 5A,B), indicating suppressed energy production. Concurrently, gene set enrichment analysis (GSEA) demonstrated statistically significant suppression (*P* < 0.05) of OXPHOS pathways following both treatments (Fig. 5C,D). Next, treatment with ionomycin (2 μg·mL^−1^) for 24 h reduced the protein levels of NDUFB8 (Complex I), COX IV (Complex IV), and ATP6 (Complex V) in Jurkat T cells (Fig. 5E). Furthermore, we measured mitochondrial ATP production and oxygen consumption rate (OCR). The results showed that treatment with ionomycin for 2 h did not cause significant changes in ATP levels or OCR. However, after prolonged treatment for 24 h, ionomycin reduced both ATP levels and OCR, an effect similar to that of AMA (Fig. 5F,G). This indicates a delayed decline in mitochondrial oxidative phosphorylation after the inhibition of mitochondrial gene transcription by ionomycin.

### Ionomycin restricts cancer cells proliferation

**Fig. 6:**
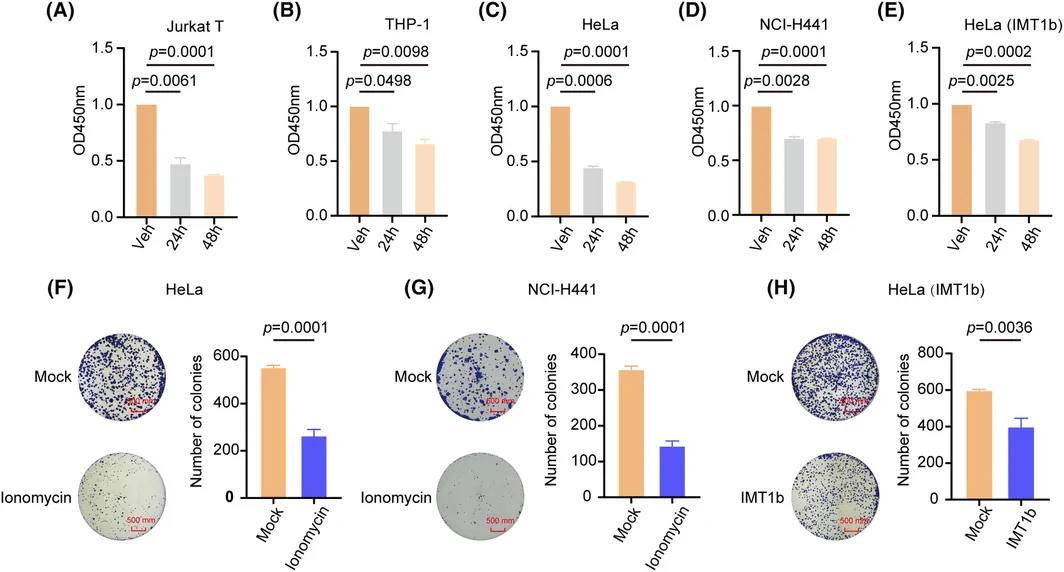
Ionomycin exhibits potent anticancer activity. (A–E) CCK‐8 analysis of viability in Jurkat T cells (A), THP‐1 (B), HeLa (C), and NCI‐H441 (D) with ionomycin (2 μg·mL^−1^) treatment for 24 h and 48 h. Effects of IMT1b (1 μg·mL^−1^) on the viability of HeLa (E) “Veh” denotes the vehicle control group. (F–G) Effects of ionomycin (2 μg·mL^−1^) on colony formation in HeLa (F) and NCI‐H441 (G) cells. (H) Effects of IMT1b (1 μg·mL^−1^) on colony formation in HeLa cells. (mean ± SD, *n* = 3 biological replicates, two‐sided Student's *t*‐test).

Next, the potential role of ionomycin in anticancer cells was explored. Previous studies indicate that while cancer cells rely on glycolysis to meet energy demands for rapid proliferation, mitochondrial OXPHOS remains crucial for tumorigenesis [3]. Different cancer types exhibit varying OXPHOS dependence [24, 25]. To determine whether ionomycin‐mediated suppression of mitochondrial‐encoded OXPHOS proteins inhibits cancer cells, we treated four cancer cell lines (Jurkat T, THP‐1, HeLa, and NCI‐H441 cells) with either ionomycin (2 μg·mL^−1^) or the established mitochondrial transcription inhibitor IMT1b (1 μg·mL^−1^). Notably, IMT1b (1 μg·mL^−1^) reduced cell viability (Fig. 6E) and colony formation capacity (Fig. 6H) in HeLa cells. Significantly, ionomycin treatment similarly reduced cell viability (Fig. 6A–E) and colony formation across all four cell lines (Fig. 6F–H and Fig. S2J,K), demonstrating even stronger effects on cancer cells. These findings provide novel insights into the role of ionomycin in cancer cell lines.

## Discussion

Mitochondria are the energy factories of the cell. They produce ATP through oxidative phosphorylation (OXPHOS). This process requires 13 key protein subunits encoded by the mitochondrial DNA (mtDNA) itself [58]. These subunits combine with others encoded by nuclear DNA to form the OXPHOS complexes. The mitochondrial RNA polymerase (POLRMT) is the first and only key enzyme that initiates mitochondrial gene expression. It transcribes all mRNA, rRNA, and tRNA encoded by mtDNA [59]. Therefore, it directly controls the maximum capacity for mitochondrial protein synthesis and energy production.

Research on cancer metabolism dates back to the early 20th century, when Otto Warburg first discovered that malignant cells preferentially obtain energy through glycolysis even under oxygen‐rich conditions, a phenomenon later termed the ‘Warburg effect’ [60]. Notably, although the Warburg effect holds a prominent position in cancer metabolism, the OXPHOS pathway also plays a significant role in the development and progression of various malignancies and may represent a potential therapeutic target [61]. More evidence indicates that certain cancers are highly dependent on OXPHOS for their energy metabolism [62]. Recent studies have shown that inhibition of OXPHOS can effectively target these specific cancer subtypes. For instance, leukemia and lymphoma, despite exhibiting glycolytic activity, are able to maintain functional OXPHOS [63]; a similar phenomenon has been observed in pancreatic ductal adenocarcinoma, where OXPHOS remains active in this aggressive cancer type [64]. Meanwhile, an increasing number of OXPHOS inhibitors have been employed in cancer therapy, including electron transport chain inhibitors (metformin, rotenone, and oligomycin) [65, 66, 67], tricarboxylic acid (TCA) cycle inhibitors (CPI‐613) [68], and antibiotics that inhibit mitochondrial protein translation (tetracycline and doxycycline) [69, 70].

Consequently, inhibition of mitochondrial transcription has recently emerged as a novel anticancer strategy [26]. Numerous studies have examined the relationship between mitochondrial DNA copy number and cancer, revealing that dysregulation of mitochondrial transcription is closely associated with many ‘hallmarks’ of cancer [71]. Small molecule compounds targeting POLRMT (IMT1 and IMT1b) have shown promising potential in cancer therapy. IMT1b is a known inhibitor of POLRMT that exhibits minimal impact on the activity of nuclear RNA polymerases (Pol I, II, and III). They can induce ATP depletion and trigger an energy crisis in colorectal cancer (CRC) and osteosarcoma (OS) cells, thereby suppressing cell proliferation [27, 29]. This selective inhibition of mitochondrial transcription underpins its value both as a research tool and a potential therapeutic agent.

In CRC cells, mitochondrial Ca^2+^ may regulate the phosphorylation of TFAM, thereby affecting its stability and promoting mitochondrial biogenesis [72]. Moreover, phosphorylated TFAM can be degraded by the mitochondrial Lon protease [73]. Therefore, we speculate that ionomycin, as a Ca^2+^‐specific ionophore, may alter mitochondrial Ca^2+^ levels, thereby influencing TFAM protein stability and ultimately affecting mitochondrial gene transcription. However, the specific molecular mechanism of how ionomycin regulates mitochondrial transcription requires further experimental investigation.

The mitochondrial ‘pearling’ phenotype is primarily triggered by Ca^2+^ signaling, whereby ER–mitochondria contact sites (ERMCSs) facilitate Ca^2+^ influx into mitochondria via the mitochondrial calcium uniporter (MCU) [44]. This process reduces membrane bending elasticity or increases membrane tension, thereby disrupting the balance between membrane tension and elasticity and inducing the beaded morphology [74]. Importantly, this morphological transition not only alters mitochondrial structure but also regulates the spatial distribution of mitochondrial DNA nucleoids [75]. In this study, we observed that ionomycin treatment induces a transition from a tubular mitochondrial network to a punctate morphology in Jurkat T cells. One plausible explanation is that this morphological remodeling resembles a mitochondrial ‘pearling’ phenomenon and could alter the spatial organization of mitochondrial nucleoids and consequently affect the efficiency of the mitochondrial transcriptional machinery. However, the precise nature of this morphological change and its functional consequences on mitochondrial transcription remain to be further investigated. Furthermore, we cannot exclude the possibility that prolonged alterations in mitochondrial homeostasis may eventually affect oxidative phosphorylation [76, 77]. In the present study, analysis of Nascent RNA‐seq data revealed that the transcriptional inhibitory effect of ionomycin is specific to mitochondrial transcription. This finding could be further validated by analyzing mitochondrial transcription in isolated mitochondrial preparations rather than in whole‐cell extracts.

Meanwhile, PMA and ionomycin were often used in combination for T‐cell activation [78, 79]. Activated T lymphocytes undergo rapid proliferation. To meet this demand, T cells undergo substantial metabolic reprogramming upon activation. In brief, resting T lymphocytes primarily rely on OXPHOS to fulfill their energy requirements, whereas activated T lymphocytes exhibit elevated glycolysis and reduced OXPHOS [80, 81, 82]. Here, we reveal from a new perspective that treatment of cells with ionomycin suppresses mitochondrial transcription, ultimately impairing OXPHOS levels. This finding may help explain the metabolic changes observed in T lymphocytes upon activation with PMA and ionomycin.

In summary, targeting mitochondrial transcription represents a novel and promising strategy for modulating cancer cell metabolism. Unexpectedly, we found that ionomycin, a classical calcium ionophore, potently suppresses overall mitochondrial RNA transcription (including rRNAs, tRNAs, and mRNAs) in cancer cells (Jurkat T, THP‐1, HeLa, and NCI‐H441 cells), impairs OXPHOS, and ultimately inhibits cancer cell proliferation.

## Conflict of interest

The authors declare no conflict of interest.

## Author contributions

XCB conceived the project. XCB, LSW, MQD, and DMH designed the experiments. LSW and MQD performed the experiments. DMH, LSW, and MQD analyzed the data. LSW and MQD wrote the manuscript. XCB, LSW, MQD, DMH, DQL, DJL, and DYZ edited manuscript. XCB, PT, ZHZ, and MHY reviewed manuscript contents. XCB supported this study financially. The authors read and approved the final manuscript.

## Supporting information


**Fig. S1.** (A) Mitochondrial Ca^2+^ levels were assessed by flow cytometry in Jurkat T cells following treatment with ionomycin (2 μg·mL^−1^) for 2 h. Treatment with Ca^2+^ UP for 0.5 h was used as a positive control. (B) Mitochondrial ROS production was assessed by flow cytometry in Jurkat T cells following treatment with ionomycin (2 μg·mL^−1^) for 2 h. Treatment with Antimycin A (AMA, 50 μg·mL^−1^) for 0.5 h was used as a positive control. (C) Mitochondrial membrane potential was assessed by flow cytometry in Jurkat T cells following treatment with ionomycin (2 μg·mL^−1^) for 2 h. Treatment with CCCP (10 mm) for 0.5 h was used as a positive control.
**Fig. S2.** (A) Effect of ionomycin (2 μg·mL^−1^) on mitochondrial morphology visualized by SIM^2^ fluorescence microscopy. (B) Effect of ionomycin (2 μg·mL^−1^) on mitochondrial DNA content, quantified by *COX3* levels. (C) Cell cycle distribution of Jurkat T cells treated with ionomycin (2 μg·mL^−1^) for 2 h, analyzed by propidium iodide (PI) staining and flow cytometry. (D) Following inhibition of cellular transcription with Actinomycin D (Act D, 10 μg·mL^−1^), the effect of ionomycin on the levels of mitochondrial genes *CYTB* was evaluated. (E) Following inhibition of mitochondrial transcription with the mitochondrial transcription inhibitor IMT1b (1 μg·mL^−1^), the effect of ionomycin on the levels of mitochondrial genes *CYTB* was assessed. (F) Jurkat T cells were treated with the nucleoside analog 5‐ethynyluridine (EU) for 16 h to label transcribed RNA, followed by Actinomycin D treatment for 2 h to inhibit RNA transcription. Cells were then either treated with ionomycin (2 μg·mL^−1^) for 16 h or treated with DMSO. Total RNA was extracted, biotinylated via click chemistry, and subjected to RNA pull‐down, after which the levels of mitochondrial genes (*COX3*, *CYTB*, *RNR1*, *RNR2*) were analyzed by qPCR. (G) Schematic diagram of the nascent RNA‐qPCR assay: cells were either untreated or treated with ionomycin (2 μg·mL^−1^) for 2 h, followed by a 15 min 5‐ethynyl uridine (5‐EU) labeling, RNA was extracted using TRNzol Reagent, biotinylated via click chemistry, pulled down with streptavidin magnetic beads, reverse‐transcribed into cDNA, and analyzed by quantitative PCR. (H) Boxplot analysis of rDNA expression changes in Jurkat T cells after treatment with ionomycin (2 μg·mL^−1^) alone or PMA and ionomycin co‐treatment mitochondrial for 2 h. (I) Effects of treatment with PMA (50 ng·mL^−1^) alone, ionomycin (2 μg·mL^−1^) alone, or co‐treatment with PMA (50 ng·mL^−1^) and ionomycin (2 μg·mL^−1^) on mitochondrial gene expression. (mean ± SD, *n* = 3 biological replicates, two‐sided Student's *t*‐test, *P < 0.05, **P < 0.01, ***P < 0.001, ****P < 0.0001.) (J, K) Effects of ionomycin (2 μg·mL^−1^) treatment on colony formation in Jurkat T (J) and THP‐1 (K) cells.
**Fig. S3.** (A) Raw data for western blotting in Fig. 1. (B) Raw data for western blotting in Fig. 5.

**Table S1.** Nascent RNA sequence results after ionomycin treatment.

**Table S2.** Mitochondrial gene reads after ionomycin treatment.

**Table S3.** Nascent RNA sequence results after PMA and ionomycin treatment.

**Table S4.** Mitochondrial gene reads after PMA and ionomycin treatment.

## Data Availability

The sequencing data generated in this study have been deposited in the GEO database under accession code GSE309432. Supporting data are also available in the Supporting Information of this article or from the corresponding author (bao_xichen@gibh.ac.cn) upon reasonable request.
