# Supplementary material for: Ionomycin suppresses cancer cell growth by disrupting mitochondrial transcription
Source: FEBS Open Bio. 2026 Sep 1:10.1002/2211-5463.70297. Online ahead of print. doi: 10.1002/2211-5463.70297 (PMC13533303; doi:10.1002/2211-5463.70297)
Supplement: Supplementary file 1 [file FEB4-9999-0-s002.pdf]

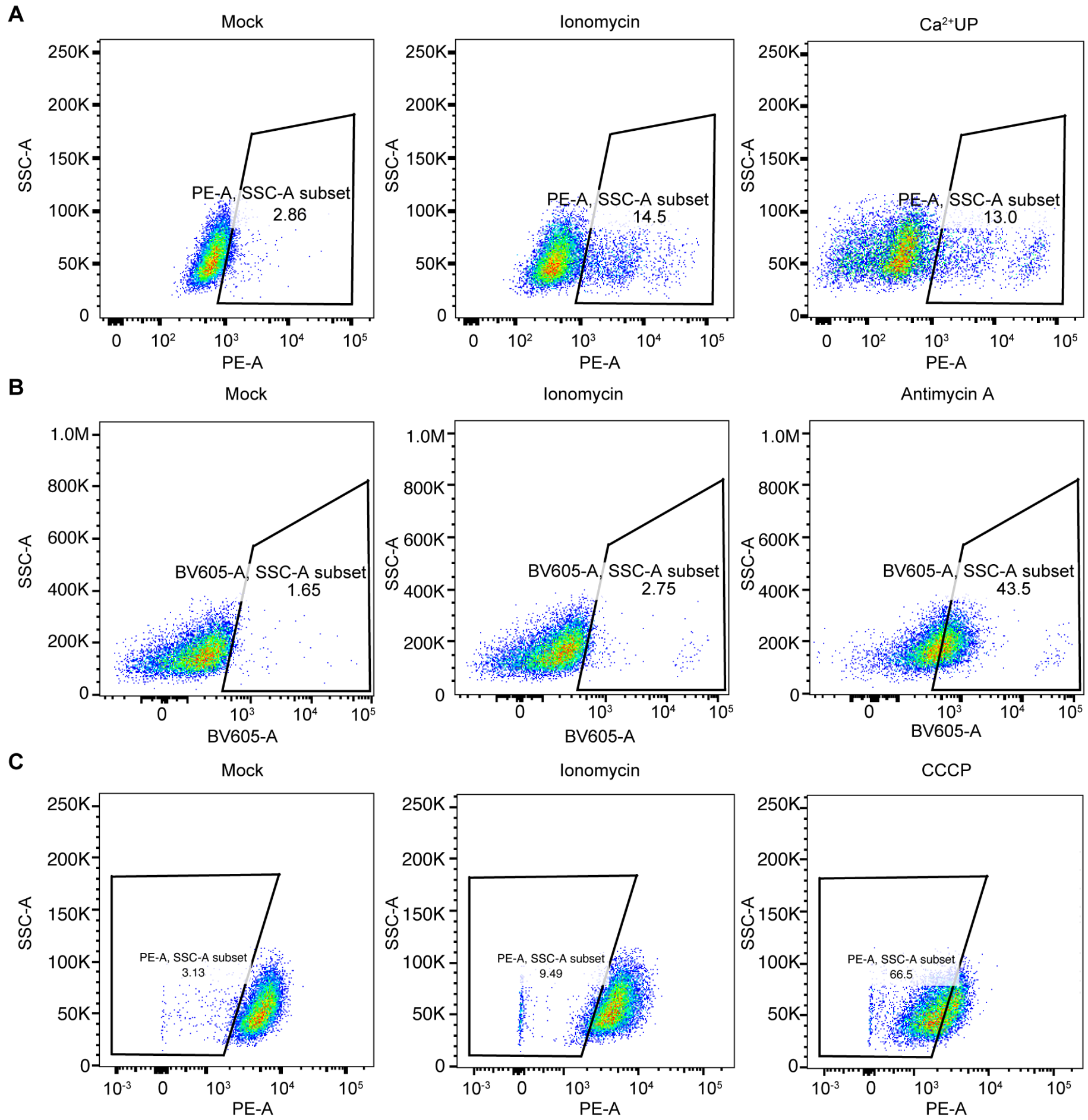

Fig. S1 A Mitochondrial Ca^{2+} levels were assessed by flow cytometry in Jurkat T cells following treatment with Ionomycin (2 µg/mL) for 2 h. Treatment with Ca^{2+} UP for 0.5 h was used as a positive control. **B** Mitochondrial ROS production was assessed by flow cytometry in Jurkat T cells following treatment with Ionomycin (2 µg/mL) for 2 h. Treatment with Antimycin A (AMA, 50 µg/mL) for 0.5 h was used as a positive control. **C** Mitochondrial membrane potential was assessed by flow cytometry in Jurkat T cells following treatment with Ionomycin (2 µg/mL) for 2 h. Treatment with CCCP (10 mM) for 0.5 h was used as a positive control.

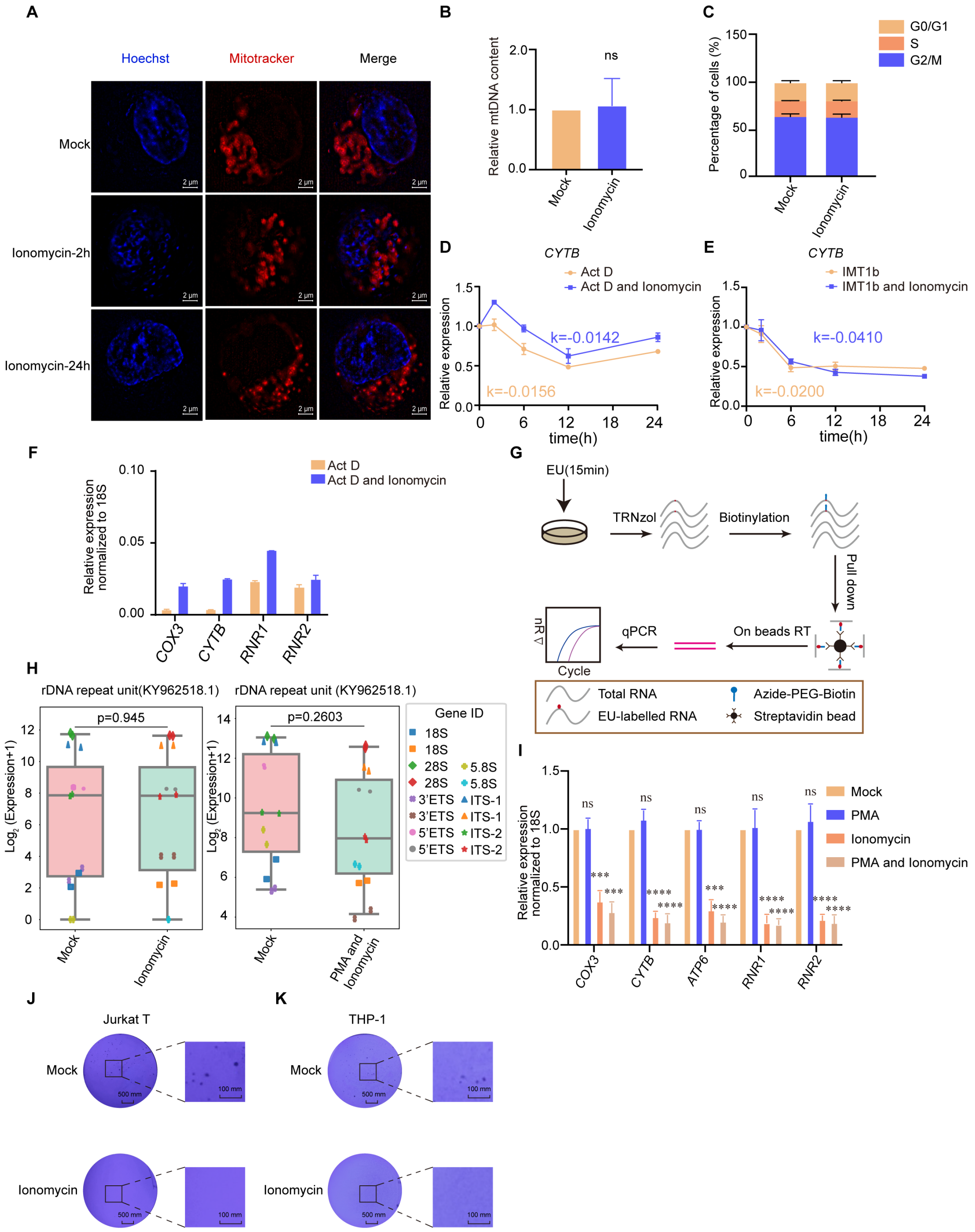

Fig. S2 **A** Effect of Ionomycin (2 μg/mL) on mitochondrial morphology visualized by SIM² fluorescence microscopy. **B** Effect of Ionomycin (2 μg/mL) on mitochondrial DNA content, quantified by *COX3* levels. **C** Cell cycle distribution of Jurkat T cells treated with Ionomycin (2 μg/mL) for 2 h, analyzed by propidium iodide (PI) staining and flow cytometry. **D** Following

inhibition of cellular transcription with Actinomycin D (Act D, 10 μg/mL), the effect of Ionomycin on the levels of mitochondrial genes *CYTB* was evaluated. **E** Following inhibition of mitochondrial transcription with the mitochondrial transcription inhibitor IMT1b (1 μg/mL), the effect of Ionomycin on the levels of mitochondrial genes *CYTB* was assessed. **F** Jurkat T cells were treated with the nucleoside analog 5-ethynyluridine (EU) for 16 h to label transcribed RNA, followed by Actinomycin D treatment for 2 h to inhibit RNA transcription. Cells were then either treated with Ionomycin (2 μg/ml) for 16 h or treated with DMSO. Total RNA was extracted, biotinylated via click chemistry, and subjected to RNA pull-down, after which the levels of mitochondrial genes (*COX3*, *CYTB*, *RNR1*, *RNR2*) were analyzed by qPCR. **G** Schematic diagram of the nascent RNA-qPCR assay: cells were either untreated or treated with Ionomycin (2 μg/mL) for 2 h, followed by a 15 min 5-ethynyl uridine (5-EU) labeling, RNA was extracted using TRNzol Reagent, biotinylated via click chemistry, pulled down with streptavidin magnetic beads, reverse-transcribed into cDNA, and analyzed by quantitative PCR. **H** Boxplot analysis of rDNA expression changes in Jurkat T cells after treatment with Ionomycin (2 μg/ml) alone or PMA and Ionomycin co-treatment mitochondrial for 2 h. **I** Effects of treatment with PMA (50 ng/mL) alone, Ionomycin (2 μg/mL) alone, or co-treatment with PMA (50 ng/mL) and Ionomycin (2 μg/mL) on mitochondrial gene expression. (mean ± SD, n = 3 biological replicates, two-sided Student's *t*-test, *$p < 0.05$, **$p < 0.01$, ***$p < 0.001$, ****$p < 0.0001$.) **J**, **K** Effects of Ionomycin (2 μg/ml) treatment on colony formation in Jurkat T (J) and THP-1 (K) cells.

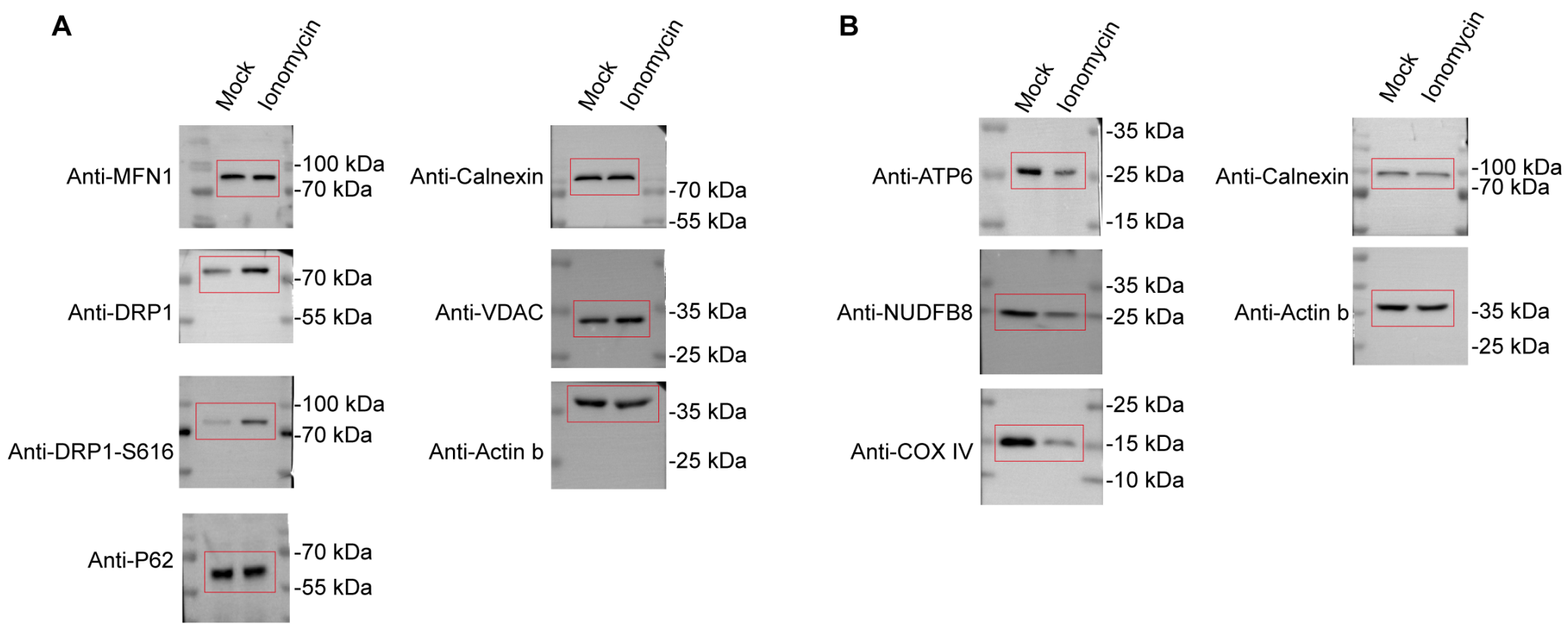

Fig. S3 A Raw data for Western Blotting in Fig. 1. **B** Raw data for Western Blotting in Fig. 5.
